# Oral leukoplakia; a proposal for simplification and consistency of the clinical classification and terminology

**DOI:** 10.4317/medoral.23372

**Published:** 2019-10-27

**Authors:** Isaäc van der Waal

**Affiliations:** 1 VU University Medical Center (VUmc)/Academic Centre for Dentistry Amsterdam (ACTA), Department of Oral and Maxillofacial Surgery and Oral Pathology, P.O. Box 7057, 1007 MB Amsterdam, The Netherlands

## Abstract

There is a distinct lack of uniformity in the definitions and clinical terminologies related to oral leukoplakia and leukoplakialike lesions and disorders. Proposals have been put forward to subclassify leukoplakia into a homogeneous and a non-homogeneous type based on color only, being either predominantly white or mixed white-and-red, respectively, irrespective of the texture of the lesion. In this proposal there is no need anymore to regard the poorly defined proliferative verrucous leukoplakia as a separate entity. Since keratosis is primarily a histopathological term, its clinical use is discouraged. Alternative terminology for these so-called keratotic lesions and disorders has been put forward. Finally, a suggestion has been made to rename the term hairy leukoplakia, being a well defined, not potentially malignant disorder particularly related to HIV-infection, into 'EBV-positive white lesion of the tongue' (EBVposWLT).

** Key words:**Potentially malignant oral disorders, oral leukoplakia, oral keratosis, hairy leukoplakia.

## Introduction

Most, if not all oral squamous cell carcinomas are preceded by clinically visible changes of the oral mucosa. Such changes are often predominantly white, being designated as leukoplakias. At present, oral leukoplakia has been defined as "A predominantly white plaque of questionable risk having excluded (other) known diseases or disorders that carry no increased risk for cancer" (1). It has been added that leukoplakia is primarily a clinical term and has no specific histology.

There are many, statistically somewhat useful, predictive factors of malignant transformation. These factors include a.o. the clinical subtype (homogeneous versus non-homogeneous), the size of the lesion, the oral subsite, and the histopathological presence or absence of epithelial dysplasia. However, these predictive factors are not reliable for use in the individual patient. This also applies to the histopathological findings and the various molecular markers that have been reported in the past decades as predictive markers of malignant transformation (2).

At present, there is a confusing use of clinical terminologies related to oral leukoplakia. Therefore, proposals will be put forward for simplification and consistency of these terminologies.

## Clinical classification and terminology

- Homogeneous and non-homogeneous leukoplakia

1. Present classification and terminology

The term homogeneous leukoplakia is by some applied for leukoplakias that are thin and flat (1), while others also recognize a thick type of homogeneous leukoplakia (3). In addition, various subvariants of homogeneous leukoplakia have been described, such as velvetlike type and pumice-stone type.

Non-homogeneous leukoplakia has traditionally been subdivided into a mixed red-and-white type (erythroleukoplakia), being often subdivided into a speckled, granular or nodular type. Verrucous leukoplakia has also been classified as a subtype of non-homogeneous leukoplakia (4).

In the past decades a separate, third, clinical subtype, not specifically being categorized as either homogeneous or non-homogeneous leukoplakia, has been introduced in the literature, being referred to as proliferative verrucous leukoplakia (PVL) (5). Proliferative verrucous leukoplakia has remained a source of confusion since its first description, due to the lack of a proper definition (6,7).

2 Proposed classification and terminology

**Figure 1:**
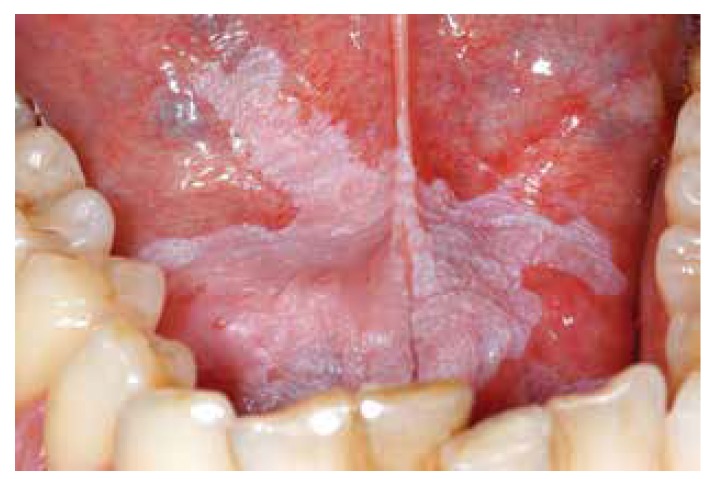
Homogeneous leukoplakia (thin, flat type).

**Figure 2:**
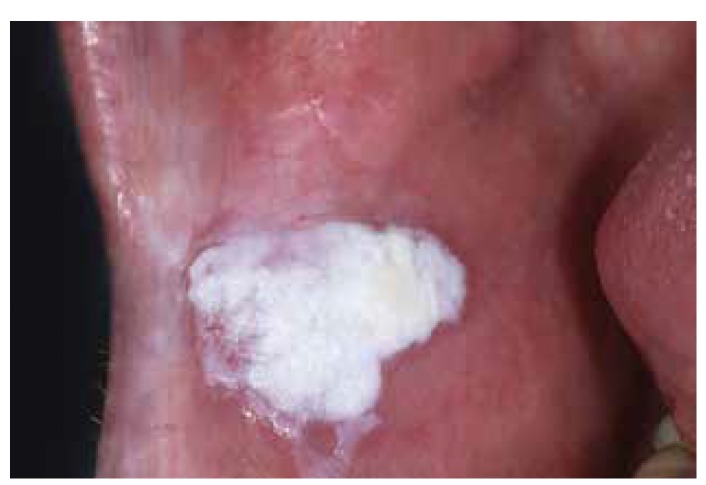
Homogeneous leukoplakia (thick or verrucous type).

**Figure 3:**
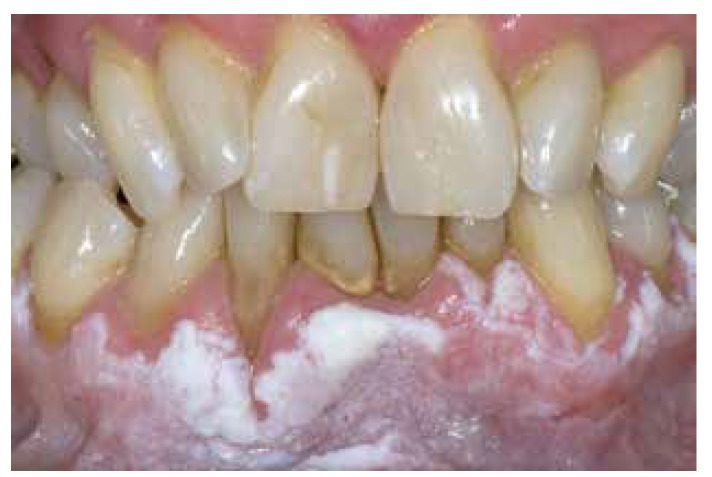
Homogeneous leukoplakia (thick or verrucous type).

It may be useful to define homogeneous primarily based on a predominantly white color, irrespective of the texture and irrespective of the extent of the disease. As a result one may recognize a thin, thick and verrucous subtype of homogeneous leukoplakia, if of relevance at all (Fig. 1, Fig. 2, Fig. 3).

Since large, widespread leukoplakias, not necessarily PVLs, carry a distinct risk of malignant transformation (8), there is no need to classify PVL as a separate entity. Being usually predominantly white, PVL can thus be classified as a homogeneous leukoplakia (Table 1).

**Table 1:**
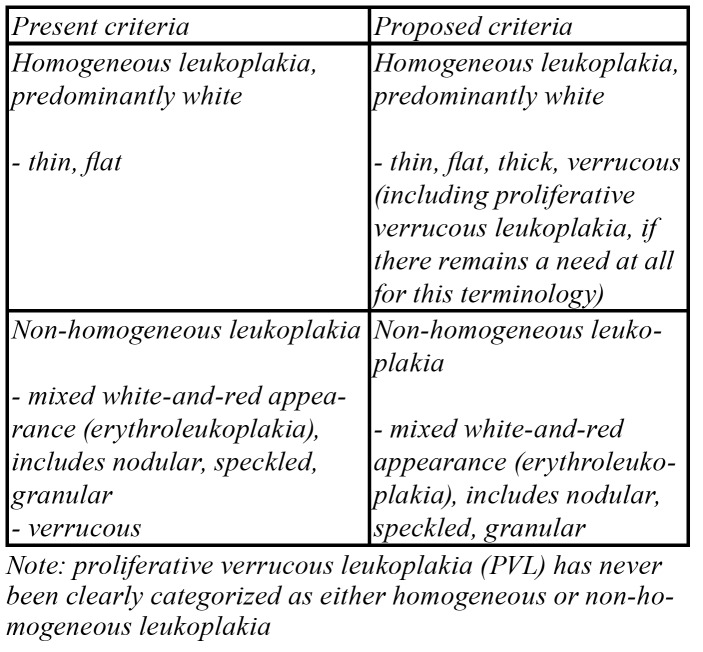
Proposal for modified criteria of homogeneous and non-homogeneous leukoplakia.

In fact, the use of the term PVL should be discouraged.

**Figure 4:**
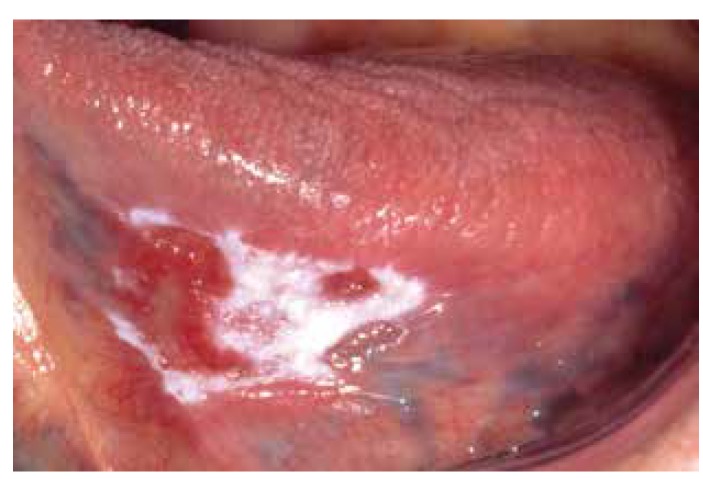
Non-homogeneous leukoplakia ('Erythroleukoplakia').

Non-homogeneous leukoplakia is primarily based on a mixed white-and-red appearance, irrespective of the texture (Fig. 4).

**Figure 5:**
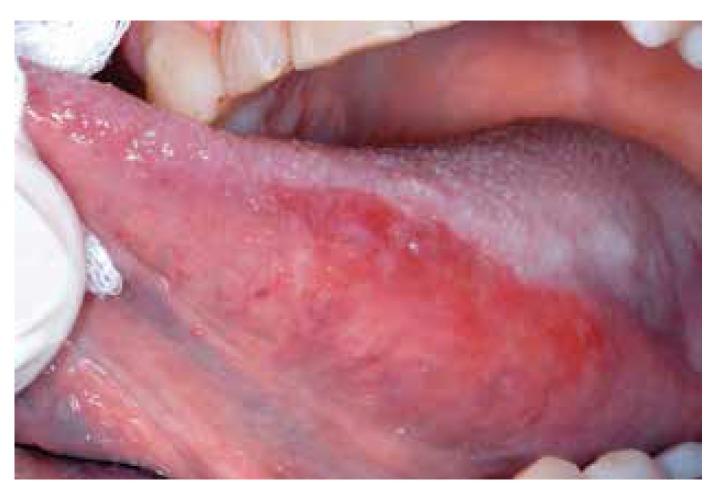
Erythroleukoplakia.

At the other end of the spectrum one recognizes erythroplakia, being predominantly red, irrespective of the texture of the lesion (Fig. 5).

When a biopsy has been taken, one may or may not encounter epithelial dysplasia, resulting in a terminology of dysplastic and a non-dysplastic leukoplakia. In case of carcinoma in situ, squamous cell carcinoma and verrucous carcinoma, the term leukoplakia is replaced accordingly.

## Keratoses

- Present classification and terminology

The term keratosis is probably quite commonly used, particularly by clinicians, to describe an oral white lesion that is otherwise not recognized as a known entity.

1. The term reversed smoking keratosis is used to describe initially white changes of the palatal mucosa due to the habit of reversed smoking. These palatal lesions carry a high risk of malignant transformation (9).

2. Sublingual keratosis refers to widespread whitish changes of the floor of the mouth, the ventral aspect of the tongue and the lingual mucosa. Apparently, sublingual keratosis carries a high risk of malignant transformation (10).

3. A few papers have been published on alveolar ridge keratosis (11,12). Apparently, the lesion is caused by chronic frictional (masticatory) trauma to the maxillary and mandibular alveolar ridges, particularly in the retromolar pad and edentulous parts of the alveolar ridges. Histopathologically, almost all of these lesions show hyperkeratosis without epithelial dysplasia. The authors suggested to remove this lesion from the category of oral leukoplakia because of its low risk of malignant transformation.

4. The term frictional keratosis has been used for white lesions on the facial attached gingiva that are supposedly caused by friction, ie. vigorously brushing of the teeth or masticatory function (13). The authors suggested to remove this lesion from the category of leukoplakia because of its low risk of malignant transformation.

5. In sanguinaria-associated keratosis the white changes of the oral mucosa, particularly located in the buccal aspect of the maxilla or the maxillary vestibule, are ascribed to sanguinaria, a herbal extract used in dentifrices and mouthrinses (14). In some cases epithelial dysplasia was observed.

6. In tobacco pouch keratosis, also referred to as snuff dipper's lesion, the clinical aspect may vary from a white to a more greyish aspect of the oral mucosa in direct contact with the tobacco product. Epithelial dysplasia is rarely encountered. The risk of malignant transformation seems to be mainly related to the type of the chewing product (15).

7. The term keratosis of unknown significance (KUS) is a histopathological diagnosis and is not used as a clinical term (16).

- Proposed classification and terminology

The use of keratosis as a clinical term is not quite appropriate. Keratosis is primarily a histopathological term that is used to describe hyperorthokeratosis or hyperparakeratosis. In addition, it has been shown that thickening of the keratin layer of the epithelium may not always be the primary factor in causing an intraoral lesion to appear white (17). Another reason to abandon keratosis as a clinical term is its likely perception by clinicians of being a benign lesion, while some of these lesions do have malignant potential.

1. The term reversed smoking associated keratosis could perhaps be renamed as reversed smoking associated (erythro)leukoplakia, but is seems better to abandon the term and to list reversed smoking under the umbrella of tobacco habits as a possible cause of leukoplakia.

2. The term sublingual keratosis actually represents an extensive leukoplakia of the floor of the mouth and, at present, there is no reason to typify leukoplakias by their oral subsite. Therefore, this terminology can be abandoned.

3. Although leukoplakias of the maxillary and mandibular alveolar ridges may carry a lower risk of malignant transformation compared with leukoplakias of the tongue or the floor of the mouth, it does not seem justified to designate alveolar ridge keratosis as a benign lesion and to remove this lesion from the category of oral leukoplakia. As in sublingual keratosis, there is at present no reason to classify leukoplakias by their oral subsite. Altogether, this terminology can be abandoned.

4. For reasons mentioned before, the term frictional keratosis might be changed into frictional lesion, but only as a provisional clinical term. When the lesion does not regress after elimination of the friction there is no justification anymore for its use and in such cases a diagnosis of leukoplakia seems preferable. There seems no need to add that the leukoplakia is located at the facial attached gingiva.

5. The use of the term sanguinaria-associated keratosis is actually based on one study of ten patients. No follow-up data were available. Therefore, the proof of sanguinaria as being the causative factor is more or less lacking. Altogether the use of the term sanguinaria-associated keratosis can perhaps be abandoned. Sanguinaria might be included in the list of possible etiological factors of leukoplakia.

6. The terms tobacco pouch keratosis and snuff dipper's lesion, can perhaps be abandonned. The role of smokeless tobacco could be specifically mentioned in the list of tobacco habits as an etiologic factor of leukoplakia.

7. Since the term keratosis of unknown significance (KUS) is a histopathological diagnosis there is no reason for any modification.

All the above is summarized in Table 2.

**Table 2:**
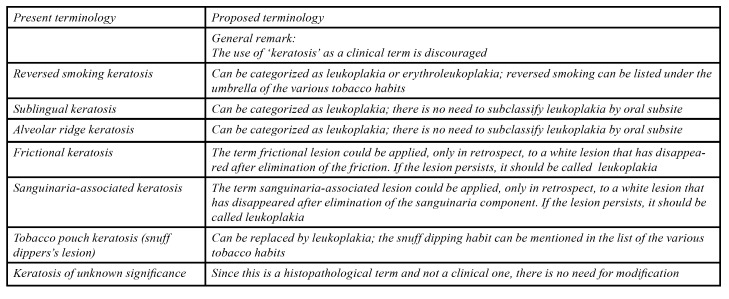
Proposal for modified terminologies of so-called keratoses.

## Hairy leukoplakia

- Present terminology

Hairy leukoplakia (HL) represents a benign whitish change of the mucosa of the borders of the tongue that mainly occurs in HIV infected patients. Nevertheless, HL may also occur as a side-effect of prolonged use of immunosuppressive drugs, e.g. after solid organ transplantation. HL rarely occurs in immunocompetent patients (18). The immunohistochemical presence of Epstein Barr virus (EBV) in superficial koilocytic epithelial cells is required for a final diagnosis of HL.

- Proposed terminology

The term 'hairy leukoplakia' is not appropriate for two reasons, being 1) it is a well defined, known, entity and, therefore does not comply with the definition of leukoplakia, and 2) it is not a potentially malignant disorder. Admittedly, it is difficult to come up with a better term. One might think of 'EBV-positive lesion of the tongue' (EBVposWLT).

## Discussion and conclusions

The present definition of leukoplakia is not much different from the ones published in 1978 and 1996 (4,19). Unfortunately, the definition is still worded in a negative way by exclusion of other white lesions.

In the present proposed classification of homogeneous and non-homogeneous leukoplakia, primarily based on color, irrespective of the texture of the lesion, the question may arise on how much redness is required for classifying a mucosal lesion as non-homogeneous leukoplakia/erythroleukoplakia instead of homogeneous leukoplakia. Likewise, one may ask how much redness is required for classifying a lesion as an erythroplakia instead of an erythroleukoplakia. Admittedly, such questions can at present not be solved in an objective, reproducible manner, being just based on subjective, clinical judgment. Application of artificial intelligence on standardized photographs may perhaps overcome this shortcoming. Furthermore, one may question the relevance of the morphological subtyping of oral leukoplakias. The extent of the leukoplakia may, indeed, be much more important in the prediction of malignant transformation than its morphology (8).

It is well realized that the Figures that have been used in this treatise are an extreme simplification of the wide range of clinical morphologies of leukoplakia that one encounters in the daily practice.

Proper communication between clinicians and pathologists is important, particularly in the field of oral potentially malignant disorders. For instance, some pathologists will deny a diagnosis of leukoplakia in the absence of epithelial dysplasia.

The clinical use of keratosis is discouraged. Furthermore, the use of strict diagnostic criteria is recommended for lesions or diseases for which a possible causative factor has been identified, e.g. frictional lesion. A somewhat arbitrarily chosen period of 4-8 weeks after removal of the possible cause should be taken into account, being a practical and safe time period, to await the disappearance of such lesion. If unchanged, a diagnosis of leukoplakia prevails. Additional descriptions such as friction associated, tobacco associated leukoplakia or sanguinaria associated leukoplakia do not seem to be of much relevance, if any.

The term 'hairy leukoplakia' is rather unfortunate and may be replaced by 'EBV-positive white lesion of the tongue' (EBVposWLT). Such an extensive nomenclature will perhaps not easily be accepted, but seems more appropriate than the term hairy leukoplakia.
